# Chirurgie Du Foie Et Des Voies Biliaires, Par J. Pantaloni (De Mirseille), Avec 348 Figures Dans Le Texte

**Published:** 1900-06

**Authors:** 


Chirurgie du Foie et des Voies Biliaires, par J. Pantaloni (de Marseille), avec 348 figures dans le texte. Octavo, pp. 626. Paris: Institute de Biblio graphie Scientifique, 93 Boulevard Saint-Germain. 1899.

This excellent treatise is divided into four parts. The first is devoted to operations upon the liver proper; the second, to operations upon the ligaments, vessels, etc., of the liver; the third and fourth to operations upon the biliary passages and further subdivided into general and special biliary surgery. Each chapter is edited upon a uniform plan in which is given in succession the history of the operation described, the technique of the various standard operations, the post-operative results and complications, and finally especial consideration of the question of the indications for operation in which the subject is discussed from the standpoint of the physician as well as the surgeon. The author hopes in this way to sufficiently arouse the interest and familiarise with results the practitioner who may thus be led to give his patients the benefit of early operative intervention.

The work is so replete, that to do it justice would require many pages. Even a cursory examination will convince the reader of its thoroughness and scientific value. It is a complete epitomé of hepatic and biliary surgery. The illustrations, 348 in number, have been carefully chosen, and are a striking feature of the book. In many instances the consecutive steps of an operation are so clearly depicted that a surgeon would have but little difficulty in performing an operation for the first time in an orderly and satisfactory manner. The value of the book is further enhanced by a comprehensive index of its contents, and of the illustrations. The author also makes use of the decimal classification of Dewey-Marcel-Boudouin, which permits the reader, when he wishes, to find the bibliography utilised by the author and to refer to the original works which he has availed himself of. This is supplemented at the end of the volume by the international ideological table and by comparing the numbers at the beginning of each chapter with this table the reader finds the references.

The book is an admirable one for all surgeons interested in hepatic and biliary surgery. J. P.

